# Understanding laterality disorders and the left-right organizer: Insights from zebrafish

**DOI:** 10.3389/fcell.2022.1035513

**Published:** 2022-12-23

**Authors:** Kadeen Forrest, Alexandria C. Barricella, Sonny A. Pohar, Anna Maria Hinman, Jeffrey D. Amack

**Affiliations:** ^1^ Department of Cell and Developmental Biology, State University of New York Upstate Medical University, Syracuse, NY, United States; ^2^ BioInspired Syracuse: Institute for Material and Living Systems, Syracuse, NY, United States

**Keywords:** organ laterality, left-right asymmetry, birth defects, zebrafish, cilia, *situs inversus*, heterotaxy syndrome, left-right organizer

## Abstract

Vital internal organs display a left-right (LR) asymmetric arrangement that is established during embryonic development. Disruption of this LR asymmetry—or laterality—can result in congenital organ malformations. *Situs inversus totalis* (SIT) is a complete concordant reversal of internal organs that results in a low occurrence of clinical consequences. *Situs ambiguous*, which gives rise to Heterotaxy syndrome (HTX), is characterized by discordant development and arrangement of organs that is associated with a wide range of birth defects. The leading cause of health problems in HTX patients is a congenital heart malformation. Mutations identified in patients with laterality disorders implicate motile cilia in establishing LR asymmetry. However, the cellular and molecular mechanisms underlying SIT and HTX are not fully understood. In several vertebrates, including mouse, frog and zebrafish, motile cilia located in a “left-right organizer” (LRO) trigger conserved signaling pathways that guide asymmetric organ development. Perturbation of LRO formation and/or function in animal models recapitulates organ malformations observed in SIT and HTX patients. This provides an opportunity to use these models to investigate the embryological origins of laterality disorders. The zebrafish embryo has emerged as an important model for investigating the earliest steps of LRO development. Here, we discuss clinical characteristics of human laterality disorders, and highlight experimental results from zebrafish that provide insights into LRO biology and advance our understanding of human laterality disorders.

## Introduction

**FIGURE 1:**
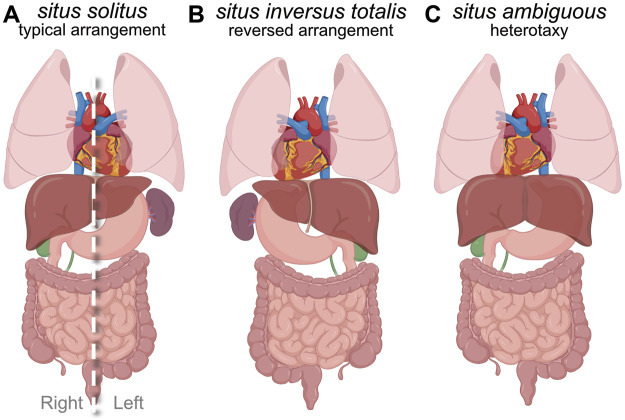
Human laterality and laterality disorders. **(A)** Diagram of *situs solitus,* which refers to the typical asymmetric development and arrangement of internal organs along the left-right body axis. Dashed line indicates the body midline. **(B)** Diagram of *situs inversus totalis,* which is a complete mirror-image reversal of organ laterality. **(C)** Diagram of one example of *situs ambiguous* or heterotaxy (other arrangement). *Situs ambiguous* is associated with a broad range of birth defects of the cardiovascular and gastrointestinal systems.

The vertebrate body plan features an asymmetric layout of internal visceral organs along the left-right (LR) body axis. In humans, the heart, stomach and spleen are typically positioned on the left side, and the liver is on the right side of the body. In addition, the left lung has two lobes and the right lung has three lobes, the developing intestines undergo LR asymmetric rotations, and the pulmonary artery and aorta connect to the right and left cardiac ventricles, respectively. This typical asymmetric arrangement of organs is referred to as *situs solitus* (Figure 1A). Interruption of developmental processes involved in setting up organ laterality during embryogenesis can result in alterations in organ placement and/or morphogenesis (Aylsworth 2001; Ramsdell 2005; Sutherland and Ware 2009; Gabriel and Lo 2020). One type of laterality disorder is a complete mirror image reversal of visceral organs, which is known as *situs inversus totalis* (SIT) (Figure 1B). There is a relatively low frequency of clinical complications associated with SIT, since a concordant reversal of organs maintains the relative spatial connections between organs. Another possibility is mixed arrangement of the thoracic and/or abdominal organs along the LR axis, which can cause a broad range of organ malformations. These defects are collectively referred to as *situs ambiguous* and are clinically classified as heterotaxy (meaning ‘other arrangement’) syndrome (HTX) (Figure 1C). HTX is characterized by a spectrum of cardiovascular and gastrointestinal birth defects that range from mild to severe enough that approximately 25% of neonates who undergo corrective procedures die during or after surgery (Buca et al., 2018). Organ laterality is a shared feature of the vertebrate body plan, and is also common in invertebrates (Speder et al., 2007; Namigai, Kenny, and Shimeld 2014; Blum and Ott 2018; Hamada and Tam 2020). Work over the last three decades has elucidated mechanisms that control laterality during embryo development, but exactly how LR spatial information is established, propagated, and maintained at molecular, cellular, and organ scales is not completely understood. In this review, we provide an overview of human laterality disorders and our current understanding of embryonic LR axis determination, and then discuss in detail how recent work using zebrafish as a model vertebrate has provided insights into the development of the embryonic left-right organizer and the earliest steps in establishing laterality.

## Human laterality disorders

### Situs inversus totalis

The incidence of SIT is reported to range between 1:10,000 and 1:30,000 births (Lin et al., 2014; Eitler, Bibok, and Telkes 2022). This condition results from reversed positioning of internal organs along the LR axis during embryonic development. It is estimated ∼3% of individuals with SIT have a congenital heart defect (Ramsdell 2005). Thus, many newborns with SIT are healthy and SIT can go undetected. In some of cases, SIT is a consequence of a broader syndrome called primary ciliary dyskinesia (PCD) (Praveen, Davis, and Katsanis 2015; Wallmeier et al., 2020; Amack, 2022). PCD is commonly caused by mutations that affect dynein arms of motile cilia (Lucas et al., 2020; Horani and Ferkol 2021; Hyland and Brody 2021). PCD patients have paralyzed cilia that cannot move mucus to clear airways (called mucociliary clearance), which leads to a buildup of mucus in craniofacial sinuses and in pulmonary airways that and ultimately affects lung function and cause bronchiectasis (Storm van’s Gravesande and Omran 2005; Leigh et al., 2019). During the first part of the 20th century, physicians Siewert (Siewert 1903) and Kartagener (Kartagener 1933) described patients with the combination of bronchiectasis and SIT, providing a link between these seemingly disparate abnormalities. Today, the triad of bronchiectasis, chronic sinusitis, and a laterality disorder is referred to as Kartagener syndrome (KS) (Leigh et al., 2009). KS is known to be caused by loss of cilia motility (Afzelius 1976) and represent a subset of PCD. More recently, it has become clear that ∼50% of PCD patients have a laterality disorder that is often SIT and, in some cases, HTX (Kennedy et al., 2007; Shapiro et al., 2014; Best et al., 2019; Nothe-Menchen et al., 2019). Most patients with SIT have a favorable prognosis and can live a normal life span.

### Heterotaxy syndrome

Heterotaxy syndrome (HTX) collectively refers to a broad spectrum of organ malformations that result from defects in establishing laterality during embryogenesis. HTX can be an isolated disorder or a feature of a genetic syndrome (Saba et al., 2022). The frequency of HTX is estimated to be ∼1 in 10,000 births, and most patients (∼90%) have a congenital heart defect (Lin et al., 2014; Eitler, Bibok, and Telkes 2022). The spectrum of congenital heart defects associated with HTX has been described in detail—including helpful diagrams and radiographs—in previous reviews (Ramsdell 2005; Maldjian and Saric 2007; Desgrange, Le Garrec, and Meilhac 2018; Agarwal et al., 2021; Saba et al., 2022). Here, we highlight cardiac and extracardiac birth defects found in HTX patients with classifications as right or left isomerism (Jacobs et al., 2007). The term isomerism refers to cases in which right-sided structures or left-sided structures are found on both sides of the body. Patients with right isomerism typically present with the most severe phenotypes on the HTX spectrum (Yi et al., 2022). These patients often have bilaterally trilobed lungs, and the absence of a spleen (asplenia) that leaves them at risk for infections (Loomba et al., 2016a; Buca et al., 2018). Characteristic cardiac defects include atrioventricular canal defects, a single functional ventricle (or large ventricular septal defect), pulmonary valve stenosis or atresia, anomalous pulmonary venous return, and transposition of the great arteries (Saba et al., 2022; Yi et al., 2022). On the other hand, individuals with left isomerism will often have bilaterally bilobed lungs and multiple spleens (polyspenia). Although the spectrum of cardiac malformations shows substantial overlap between left and right isomerism, frequent defects in left isomerism include atrioventricular septal defects, double outlet right ventricle, interrupted inferior vena cava, and atrioventricular heart block (Loomba et al., 2016b; Buca et al., 2018). It is important to note that organ arrangement in some HTX cases deviates from these descriptions of left or right isomerism (Yim et al., 2018). An extracardiac defect common in both types of isomerism is malrotation of the intestine (Ryerson et al., 2018; Saba et al., 2022). Failure of the intestine to complete an asymmetric 270° rotation around the superior mesenteric artery during development can cause mispositioning and give rise to volvulus (Langer 2017), which can disrupt blood supply and cause obstruction. Additional presentations can include biliary atresia, duodenal atresia, and tracheo-esophogeal fistula (Kothari 2014). Taken together, clinical findings indicate that the altered arrangement of organs in HTX patients creates complications in cardiopulmonary function, immune response, and digestion that can have varying presentations throughout life.

Individuals with HTX typically require medical intervention (Saba et al., 2022). In an ideal situation, a prenatal diagnosis can be made with ultrasound and/or prenatal echocardiogram that can facilitate pre-planning for clinical care after delivery. Due to varying presentation, HTX treatment depends on the severity of the birth defect. Often, intervention includes surgery in infancy, after which patients will require lifelong care. The advancement of technology and introduction of new interventions has increased the life expectancy of affected individuals (Kim 2011). As the population of patients living with congenital malformations increases, scientists and physicians are challenged to better understand the genetic and mechanistic underpinnings of these disorders while also generating more effective treatment plans over an increased life span. Advances in these areas are needed to improve long-term outcomes for patients with laterality disorders.

## Left-right patterning of vertebrate embryos

### Asymmetric nodal signaling

**FIGURE 2:**
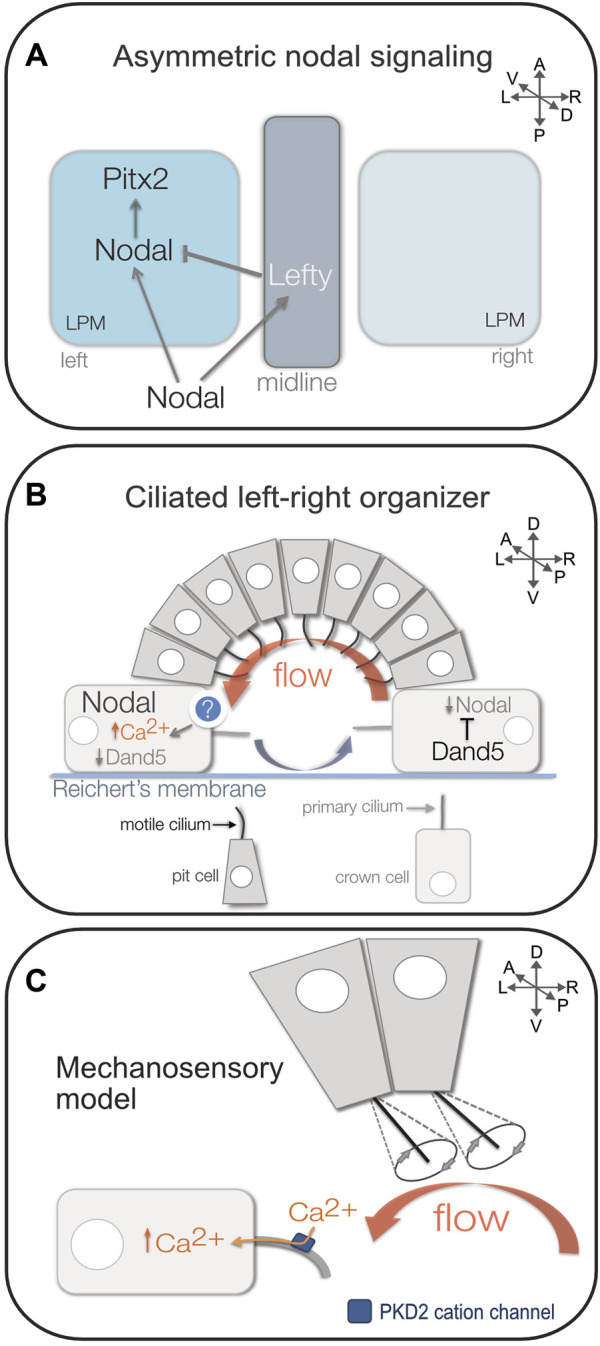
Mechanisms of vertebrate LR patterning. **(A)** Diagram representing asymmetric Nodal (TGF-β) signaling in left lateral plate mesoderm (LPM). Nodal activates its own transcription as well as the Nodal antagonist Lefty in the embryonic midline that restricts Nodal to the left side, and the transcription factor Pitx2 that can regulate asymmetric morphogenesis of organs such as the heart and gut. **(B)** Diagram of the mouse left-right organizer (referred to as the node/PNC). Epithelial pit cells with motile cilia generate a leftward fluid flow (red arrow) in a cavity covered by Reichert’s membrane. Return flow (blue arrow) occurs away from the ciliated epithelium. Leftward flow is sensed in larger crown cells that have immotile primary cilia. In left-sided crown cells, flow induces increased Ca^2+^ fluxes and degradation of the Nodal inhibitor Dand5. Loss of Dand5 increases Nodal expression in left crown cells, which can then activate asymmetric Nodal expression in left LPM. **(C)** Diagram of the mechanosensory cilia model for sensing flow. It is proposed that posteriorly tilted motile cilia on pit cells generate a leftward flow that induces bending of mechanosensory primary cilia in left-side crown cells to open the stretch-activated cation channel PKD2 in the ciliary membrane that initiates asymmetric Ca^2+^ signaling. A = anterior, P = posterior, L = left, R = right, D = dorsal, V = ventral.

The clinical impact of birth defects associated with laterality disorders has driven research efforts to identify and understand underlying causes. Copy number variant screens and genome sequencing of patients with laterality defects have provided candidate genes (Fakhro et al., 2011; Cowan et al., 2016; Hagen et al., 2016; Liu et al., 2018; Blue et al., 2022). However, due to limitations of available human genetic data and experimental resources, the field has relied on several animal models—which include chicken, frog, mouse, medaka, and zebrafish—to provide mechanistic insights into organ laterality. These models have been instrumental in advancing our understanding of the process of developing LR asymmetry, which we refer to as LR patterning, in vertebrate embryos. For over two decades, it has been recognized that vertebrate LR patterning involves asymmetric expression of a highly conserved transforming growth factor-beta (TGF-β) family secreted signaling molecule called Nodal in the left lateral plate mesoderm during early somite stages of development (Levin et al., 1995; Collignon, Varlet, and Robertson 1996; Meno et al., 1996). The Nodal signaling ligand binds type I and type II serine-threonine kinase receptors at the cell surface that then phosphorylate cytoplasmic Smad2 and/or Smad3 proteins, which facilitates their interaction with Smad4 and subsequent translocation into the nucleus to impact gene transcription (Shen 2007). In lateral plate mesoderm, Nodal signaling can activate its own transcription as well as other targets that include Lefty proteins that act as feedback inhibitors of Nodal signaling (Meno et al., 1996; Nakamura et al., 2006) and the homeobox transcription factor Pitx2 that can regulate genes involved in asymmetric morphogenesis of the heart and gastrointestinal tract (Logan et al., 1998; Piedra et al., 1998; Ryan et al., 1998; Yoshioka et al., 1998; Shiratori et al., 2001; Kurpios et al., 2008) (Figure 2A). Cellular and molecular details of this conserved asymmetric Nodal signaling pathway have been described in recent review articles (Shiratori and Hamada 2014; Belo, Marques, and Inacio 2017; Zinski, Tajer, and Mullins 2018; Hamada, 2020). Importantly, genetic analyses in animal models and humans indicates that mutations that disrupt Nodal signaling can result in organ laterality disorders (Barnes and Black 2016; Montague, Gagnon, and Schier 2018; Li et al., 2019).

### The left-right organizer

A key question in the field has been *what mechanisms break bilateral symmetry in the vertebrate embryo?* Motile cilia were associated with laterality disorders in patients with KS in the 1970s (Afzelius 1976), but when and where cilia function during LR patterning was not known. Work in the 1990s associated motile cilia in an embryonic structure referred to as the ventral node with establishing LR asymmetry of the mouse embryo (Sulik et al., 1994; Nonaka et al., 1998). Although this ciliated structure in the mouse embryo is conventionally referred to as the ‘ventral node’ or ‘node’ in the literature, a comparative analysis in the rabbit embryo found cilia and *Nodal* expression are located in the posterior notochord (PNC) that is distinct from the node (Blum et al., 2007). Since it has been proposed that the PNC is also the site of laterality determination in the mouse embryo (Blum et al., 2007), we refer to this structure as the node/PNC. The node/PNC is a transient structure during early somite stages that consists of epithelial cells that project a single cilium into a pit-like cavity filled with extraembryonic fluid and covered by a specialized basement membrane called Reichert’s membrane (Sulik et al., 1994; Lee and Anderson 2008) (Figure 2B). Central “pit cells” in the node/PNC have primarily motile cilia, whereas surrounding “crown cells” have primarily immotile cilia (McGrath et al., 2003; Yoshiba et al., 2012). Motile cilia become posteriorly tilted in response to planar cell polarity cues (Hashimoto et al., 2010; Hashimoto and Hamada 2010; Song et al., 2010) and beat with a unidirectional vortical motion (Nonaka et al., 1998; Shinohara et al., 2015). Posterior tilting of motile cilia creates an effective power stroke away from the cell surface and a recovery stroke near the surface that results in a leftward fluid flow across the node/PNC (Cartwright, Piro, and Tuval 2004; Nonaka et al., 2005; Okada et al., 2005) that is linked to asymmetric *Nodal* expression (Figures 2B,C). In a landmark study, knockout of Kif3b, a kinesin family motor protein, was found to abolish leftward flow in the mouse node/PNC due to loss of cilia, which correlated with bilateral or absent asymmetric Nodal signaling and organ laterality defects (Nonaka et al., 1998). Similar phenotypes were observed in mice with mutations that paralyze cilia, including *inversus viscerum*, which affects Dynein Axonemal Heavy Chain 11 (Dnah11; also referred to Left-Right Dynein) (Supp et al., 1997; Supp et al., 1999), and Dynein Axonemal Heavy Chain 5 (Dnah5) (Ibanez-Tallon, Gorokhova, and Heintz 2002), supporting the idea that loss of cilia-generated leftward flow alters asymmetric Nodal signaling. In complementary work, artificial flow applied across the node/PNC in cultured mouse embryos was found to be sufficient to direct laterality (Nonaka et al., 2002). Together, these studies provided evidence that cilia located in the node/PNC play an essential role in establishing asymmetric *Nodal* expression and organ laterality in the mouse embryo.

Shortly after the ciliated node/PNC was implicated in LR patterning of the mouse embryo, similar transient monociliated structures were identified in other vertebrate embryos—zebrafish, frog, and chicken—suggesting a conserved cilia-based mechanism regulates LR patterning in vertebrates (Essner et al., 2002). Functional studies confirmed the presence of motile cilia and asymmetric fluid flow in Kupffer’s vesicle in zebrafish (Essner et al., 2005; Kramer-Zucker et al., 2005) and the gastrocoel roof plate in frog (Schweickert et al., 2007). Genetic or embryological perturbation of these ciliated structures disrupted asymmetric Nodal pathway expression and organ laterality. Motile cilia and asymmetric fluid flows were also described in the posterior notochordal plate in rabbit, Kupffer’s vesicle in medaka, and the gastrocoel roof plate in axolotl embryos (Okada et al., 2005; Blum et al., 2009). These structures are now referred to as the ‘left-right organizer’ (LRO) in the embryo. Interestingly, cilia identified in Hensen’s node in the chicken embryo are likely immotile (Stephen et al., 2014), and Hensen’s node does not form a cavity to accommodate fluid flow (Manner 2001; Dathe et al., 2002). Live imaging revealed that sonic hedgehog (*Shh*) expressing cells migrate asymmetrically around Hensen’s node (known as “node rotation”) to establish left-sided asymmetric *Shh* expression and an asymmetric node morphology (Cui, Little, and Rongish 2009; Gros et al., 2009). Left-sided *Nodal* expression in lateral plate mesoderm depends on *Shh*, and has been proposed to be influenced by asymmetries in developing midline (notochord and floor plate) structures (Otto et al., 2014). Similar to chick, two mammalian embryos—pig and cow—have an asymmetric node/PNC morphology that lacks cilia and/or space for fluid flow (Gros et al., 2009; Schroder et al., 2016). Analysis of *Nodal* expression at the node/PNC in four mammals revealed two classes of embryos: bilaterally symmetric *Nodal* was observed in embryos with motile cilia and fluid flow (mouse and rabbit), whereas asymmetric (left-biased) *Nodal* expression was found in embryos that lack cilia and/or space for flow (pig and cow) (Schroder et al., 2016). Recent work with gecko and turtle embryos identified asymmetric (left-biased) *Nodal* expression and a lack of motile cilia at the blastopore (equivalent to Hensen’s node) of these embryos, suggesting LR patterning proceeds independent of cilia in reptiles (Kajikawa et al., 2020). Together, these findings challenge the idea of a single conserved mechanism for breaking LR symmetry in vertebrates and suggest that in addition to cilia-driven flows, other asymmetric cellular behaviors and/or cellular chirality may play important roles in LR patterning of vertebrate embryos (Inaki, Liu, and Matsuno 2016; McDowell, Rajadurai, and Levin 2016; Wan et al., 2016; Blum and Ott 2018; Hamada, 2020; Rahman et al., 2020).

In embryos with ciliated LROs, the exact mechanism(s) by which cilia-driven fluid flow impacts LR patterning are not fully understood. Work from mouse indicates leftward flow leads to the degradation of mRNA encoding Dand5 (also called Cerl2), a Cerberus/Dan family protein that functions to inhibit Nodal, in crown cells on the left side of the LRO (Marques et al., 2004; Nakamura et al., 2012), which results in higher Nodal activity on the left (Figure 2B). Degradation of *Dand5* mRNA is regulated by the RNA binding protein Bicc1 that binds the 3′-UTR of *Dand5* mRNA (Minegishi et al., 2021). Similar post-transcriptional regulatory mechanisms generate LR asymmetry of Dand5 homologs in fish and frog (Hojo et al., 2007; Schweickert et al., 2010; Maerker et al., 2021). A recent phylogenetic study comparing vertebrates with motile cilia in their LRO versus those without cilia elucidated a set of 5 proteins lost in animals without a ciliated LRO (Szenker-Ravi et al., 2022). Analyses of one of these proteins, a metalloprotease termed ciliated left right organizer metallopeptidase (CIROP), revealed that it functions as an upstream factor necessary for *Dand5* asymmetry in mouse, frog and fish embryos, and that *CIROP* mutations are found in human laterality patients (Szenker-Ravi et al., 2022). How flow is sensed by LRO cells to asymmetrically impact CIROP, Bicc1, and potentially other regulators remains unclear. Multiple models have been proposed for how this may happen, and these have been recently reviewed in detail elsewhere (Hamada 2016; Schweickert et al., 2017; Shinohara and Hamada 2017; Little and Norris 2021). Briefly, it has been proposed that cilia-driven asymmetric fluid flow transports either secreted signaling molecules (Nonaka et al., 1998; Okada et al., 2005) or extracellular vesicles (Tanaka, Okada, and Hirokawa 2005; Solowiej-Wedderburn et al., 2019) to the left side of the LRO to induce asymmetric calcium ion (Ca^2+^) flux and downstream signaling events. On the other hand, non-motile mechanosensory cilia may sense flow by bending to open stretch-activated ion channels in the ciliary membrane that initiate asymmetric Ca^2+^ signaling (McGrath et al., 2003; Tabin and Vogan 2003) (Figure 2C). Over the last several years, this mechanosensory cilia mechanism has been favored based on evidence that includes 1) involvement of cilia-localized PKD (polycystic kidney disease) ion channels (composed of subunits Pkd2 and Pkd1l1) in LR patterning of vertebrate embryos (Pennekamp et al., 2002; Bisgrove et al., 2005; Bataille et al., 2011; Field et al., 2011; Kamura et al., 2011), 2) a requirement for Pkd2 in non-motile cilia in the mouse LRO to respond to flow (Yoshiba et al., 2012), and 3) observation of flow-dependent asymmetric Ca^2+^ flux that initiates in immotile cilia and then propagates to cells preferentially on the left side of LROs in zebrafish and mouse embryos (Yuan et al., 2015; Mizuno et al., 2020). However, other experiments that visualized Ca^2+^ dynamics suggest non-motile LRO cilia in the mouse embryo do not function as Ca^2+^ mechanosensors (Delling et al., 2016). Thus, understanding exactly how fluid flow is translated into molecular asymmetry in the LRO—*via* chemosensing, mechanosensing, or possibly both—is an active area of investigation.

Form and function studies in mouse, frog, and zebrafish have uncovered several intriguing features of ciliated LROs. First, analyses of cell size and shape suggest the cellular architecture of the LRO is critical for its function. In frog, monociliated LRO cells are smaller than flanking endodermal cells, and there is an asymmetric distribution of polarized (posteriorly tilted) cilia along the anterior-posterior (AP) axis with a lower density of polarized cilia in posterior region (Schweickert et al., 2007). In the mouse LRO, smaller pit cells are tightly packed in the posterior region, and larger cells are found at the anterior region (Nonaka et al., 1998; Lee and Anderson 2008). This creates an AP gradient of motile cilia that impacts flow dynamics. Leftward flow has the highest velocity across the posterior pole, which correlates with a high frequency of Ca^2+^ fluxes—the first known molecular asymmetry—in the left-posterior region of the LRO (Mizuno et al., 2020). The zebrafish LRO has a similar AP gradient of ciliated cells, except that smaller cells, more cilia, and higher flow velocities are found in the anterior region (Kreiling et al., 2007; Okabe et al., 2008; Wang et al., 2011; Sampaio et al., 2014; Ferreira et al., 2017). Perturbations in mouse or zebrafish that disrupt LRO cellular architecture alter asymmetric Nodal signaling and LR patterning [reviewed in (Lee and Anderson 2008; Amack 2014; Dasgupta and Amack 2016]. Second, it appears that normal LR patterning does not depend on “full-strength” fluid flows generated by LRO cilia. In frog, paralyzing cilia on the entire right side of the LRO had no phenotypic consequence, indicating flow generated by the left side is sufficient for normal laterality (Vick et al., 2009). In the mouse LRO, mutations that paralyze cilia with variable penetrance revealed that only 2 motile cilia (out of 200–300) are needed to generate normal LR asymmetry (Shinohara et al., 2012). While these surprising results argue against a requirement for a strong flow across the entire LRO, leftward flows were maintained in both the frog and mouse experiments, and in mice with 2 motile cilia the weak flow was across the posterior LRO. Mathematical modeling and experimental perturbations in zebrafish also predict only a subset of motile cilia in the LRO is necessary to create flows needed to break symmetry (Sampaio et al., 2014; Tavares et al., 2017). A better understanding of the dynamics of weak flows may provide mechanistic insight into how the flow generates asymmetric signaling. Third, morphogenesis of the transient LRO is highly dynamic, which suggests precise developmental timing of flow creation and flow sensing is critical for generating asymmetric signals. In zebrafish, the number of motile cilia increases over developmental time in concert with cell shape changes and lumen expansion to create asymmetric flow (Oteiza et al., 2008; Wang, Manning, and Amack 2012; Yuan et al., 2015; Ferreira et al., 2017). At later somite stages of mouse development, the LRO changes shape and leftward flow is no longer detected even though cilia are still motile (Nonaka et al., 1998). Together, these findings indicate a tight temporal relationship between a dynamic LRO architecture and the generation of leftward flow and asymmetric signaling.

## Zebrafish as a model system to understand left-right patterning

Zebrafish is a useful model organism to investigate the origins, regulators, and outcomes of left-right patterning events. External fertilization and rapid embryo development allows real-time analysis of the earliest stages of LR patterning without perturbing the embryo. Transparency of the embryo facilitates spatial analysis of gene expression and protein localization, and several transgenic strains that express fluorescent proteins in specific cell types relevant to LR development are readily available. In addition, genes and pathways can be modulated using a wide range of genetic and pharmacological approaches. The combination of these features has been used successfully to identify and test candidate genes and mechanisms for laterality defects in humans, and presents opportunities to develop new technologies to further understand LR patterning.

### Zebrafish laterality

**FIGURE 3:**
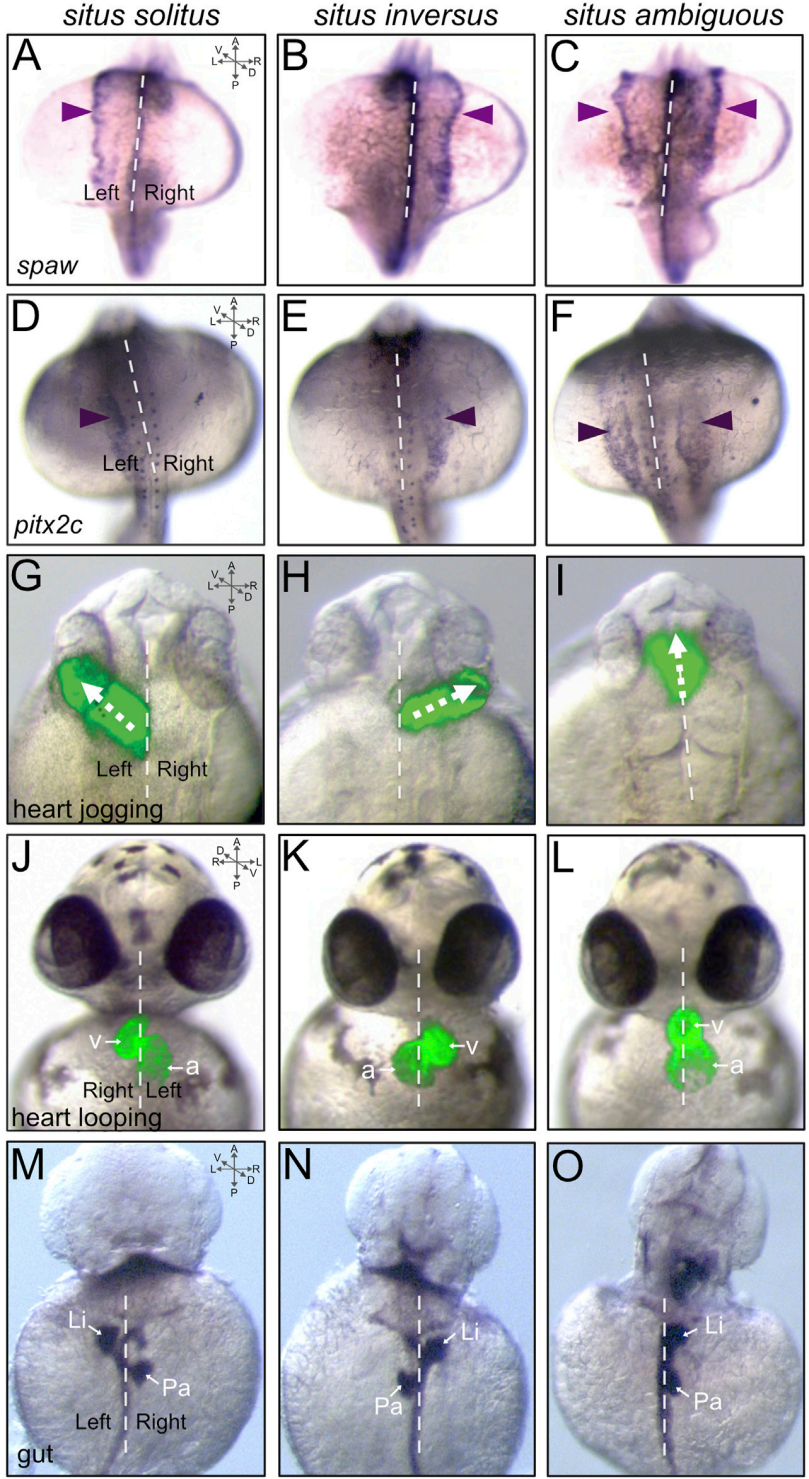
Asymmetric markers and laterality defects in the zebrafish embryo. **(A–F)** Dorsal views of RNA *in situ* hybridizations of *spaw* expression at the 18-somite stage **(A–C)** or *pitx2c* expression at the 20-somite stage **(D–F)**. Typical asymmetric expression (*situs solitus*) occurs in left lateral plate mesoderm [arrowhead in **(A,D)**]. Defects in LR patterning include right-sided [*situs inversus*; arrowhead in **(B,E)**] or bilateral [*situs ambiguous*; arrowheads in **(C,F)**] expression. **(G–L)** Visualization of heart jogging at 26 hpf **(G–I)** and heart looping at 48 hpf **(J–L)** in living transgenic *Tg(myl7:GFP)* (Huang et al., 2003) embryos that express GFP (green) specifically in cardiomyocytes. GFP fluorescence images are superimposed on brightfield images of the embryo. The forming heart tube normally migrates (‘jogs’) to left of the midline [arrow in **(G)**]. LR patterning defects can result in reversed (arrow in H) or midline [arrow in **(I)**] jogging. Next, as in other vertebrates, the heart typically loops to the right [*situs solitus*; **(J)**]. Heart looping defects can include reversed [*situs inversus*; **(K)**] and midline [*situs ambiguous*; **(L)**] looping. v = ventricle, a = atrium. **(M–O)** Dorsal view of RNA *in situ* hybridizations of *foxa3* expression in the gastrointestinal tract at 48 hpf. The marker *foxa3* labels the gut tube, left-sided liver (Li) and right-sided pancreas (Pa) in embryos with *situs solitus* **(M)**. Examples of laterality defects of the gut include reversed orientation [*situs inversus*; **(N)**] and left-sided liver [*situs ambiguous*; **(O)**]. Dashed lines indicate the midline. A = anterior, P = posterior, L = left, R = right, D = dorsal, V = ventral.

As in other vertebrates, a Nodal-related protein—called Southpaw (Spaw)—is asymmetrically activated in left-sided lateral plate mesoderm and induces left-sided expression of *nodal*, *lefty* and *pitx2* genes during LR patterning of the zebrafish embryo that will generate asymmetries in the heart, gut, and brain (Long, Ahmad, and Rebagliati 2003; Matsui and Bessho 2012; Grimes and Burdine 2017; Montague, Gagnon, and Schier 2018). Similar to mouse, fluid flow generated by motile cilia in the LRO (Kupffer’s vesicle) during early somite stages results in elevation of the Dan family Nodal inhibitor Dand5 (also called Charon in zebrafish) (Hashimoto et al., 2004) on the right side of the LRO at the 8 somite stage (Lopes et al., 2010; Schneider et al., 2010; Juan et al., 2018). Asymmetric Spaw signaling at the LRO then activates *spaw* expression in the left lateral plate mesoderm at 10–12 somite stages, which expands anteriorly between the 12–23 somite stages (Long, Ahmad, and Rebagliati 2003; Wang and Yost 2008; Montague, Gagnon, and Schier 2018) (Figure 3A). Asymmetric expression of *lefty1*, *lefty2,* and *pitx2* (*pitx2*c isoform) mRNA can be detected at 19–22 somite stages in lateral plate mesoderm, heart field, and diencephalon in the brain (Bisgrove, Essner, and Yost 1999; Thisse and Thisse 1999; Bisgrove et al., 2000; Essner et al., 2000; Campione et al., 1999). Expression of *lefty1* in the midline restricts Nodal signaling to the left lateral plate mesoderm (Bisgrove, Essner, and Yost 1999; Montague, Gagnon, and Schier 2018). Disruption of early LR patterning steps can alter *spaw* expression, such that it is right-sided (e.g., *situs inversus*) (Figure 3B) or bilaterally expressed (e.g., *situs ambiguous*) (Figure 3C). Left-sided expression of *pitx2c* (Figure 3D) is similarly altered in embryos with LR patterning defects (Figures 3E,F). It is interesting to note that mutations in *spaw* can alter LR asymmetry of the heart (Montague, Gagnon, and Schier 2018) and gut (Noel et al., 2013), but mutations in *pitx2* have no such effect on gross heart or gut laterality (Ji, Buel, and Amack 2016). Recently, defects in *spaw* asymmetry were found to be uncoupled from heart and gut laterality defects in *jnk* mutants (Derrick et al., 2022). These findings suggest pathways and/or factors in addition to the Nodal-Pitx2 signaling axis can impact heart and gut LR morphogenesis. Along these lines, Nodal-independent actin cytoskeletal dynamics (Noel et al., 2013), BMP signaling (Chocron et al., 2007; Smith et al., 2011; Lenhart et al., 2013; Veerkamp et al., 2013; Lombardo et al., 2019), and right-sided asymmetric expression of the Prrx1a transcription factor (Ocana et al., 2017; Rago et al., 2019) have been implicated in regulating laterality. However, the function of Prrx1a has recently been challenged by zebrafish mutants that have normal cardiac laterality (Castroviejo et al., 2020; Tessadori et al., 2020). Defining precise mechanisms, interactions, crosstalks, and/or compensations among converging pathways are key areas for future investigation.

Details of known cellular and molecular mechanisms that control asymmetric organogenesis in zebrafish have been reviewed previously (Bakkers, Verhoeven, and Abdelilah-Seyfried 2009; Grimes and Burdine 2017; Smith and Uribe 2021). Here, we briefly introduce these organ LR asymmetries, discuss when they develop, and describe methods to detect them. The zebrafish primitive heart tube forms between 22 and 26 h post-fertilization (hpf), and migrates (‘jogs’) to the left of the midline as it elongates (Chen et al., 1997) (Figure 3G). Imaging live transgenic embryos with fluorescently labeled cardiomyocytes revealed faster migration of left-sided cells, which is dependent on Bmp and Spaw signaling (Baker, Holtzman, and Burdine 2008; de Campos-Baptista et al., 2008; Smith et al., 2008). This process of heart jogging includes a rotation that transforms the initial left side of the heart field (e.g., marked by *lefty2* expression) into the dorsal side of the elongating heart tube (Baker, Holtzman, and Burdine 2008; Rohr, Otten, and Abdelilah-Seyfried 2008; Smith et al., 2008). Perturbation of LR patterning can result in reversed heart jogging (Figure 3H), or extension along the midline (Figure 3I). After elongation and jogging, the zebrafish primitive heart tube undergoes the highly conserved process of rightward cardiac looping that occurs across vertebrates (Campione and Franco 2016; Desgrange, Le Garrec, and Meilhac 2018) (Figure 3J). Cardiac looping morphogenesis occurs in zebrafish between 28 and 48 hpf, and relies on LR patterning cues and intrinsic activity of the actomyosin cytoskeleton (Noel et al., 2013; Montague, Gagnon, and Schier 2018). A second rotation between 28 and 30 hpf brings the dorsal side of the heart (the original left side of the heart field) back to the left side of the heart (Baker, Holtzman, and Burdine 2008). Live imaging identified a torque-like deformation at the arterial pole of the and anisotropic growth of ventricle and atrium during looping morphogenesis (Lombardo et al., 2019). Laterality defects can result in reversed heart looping (e.g., *situs inversus*) (Figure 3K) or lack of heart looping such that the heart remains symmetric along the midline (e.g., *situs ambiguous*) (Figure 3L). Although the direction of cardiac looping can be uncoupled from cardiac jogging, leftward jogging promotes robust rightward looping morphogenesis (Grimes et al., 2020).

The zebrafish gastrointestinal tract also undergoes LR asymmetric morphogenesis and endodermal organs take up asymmetric positions, with the liver and gall bladder developing on the left side and pancreas placed on the right (Figure 3M). In chicken and mouse embryos, asymmetric Pitx2 expression regulates asymmetric cellular behaviors to bend the dorsal mesentery that suspends the gut tube from the abdominal wall (Davis et al., 2008), which in turn results in looping of the intestines. Intriguing recent work indicates nodal-independent asymmetric Pitx2 activity drives a mechanical feedback mechanism in the dorsal mesentery (Sanketi et al., 2022). Experiments in mouse and frog indicate asymmetry of the stomach may not depend on extrinsic forces (e.g., mediated by dorsal mesentery), but rather depends on development of asymmetric cellular architectures directed by intrinsic laterality cues that include Nodal and Pitx2 activity (Davis et al., 2017). In zebrafish, the developing gut tube is not suspended by a dorsal mesentery, but rather receives directionality from the lateral plate mesoderm, located on either side of the endoderm (Horne-Badovinac, Rebagliati, and Stainier 2003). At 26 hpf, the lateral plate mesoderm asymmetrically migrates to forcing the gut tube to the left side of the embryo. Disruption of LR patterning signals can result in gastrointestinal laterality defects, which include reverse orientation of the gut (e.g., *situs inversus*) (Figure 3N), or *situs ambiguous* that is reminiscent of HTX in humans (Figure 3O).

Several methods are available to detect organ asymmetries in zebrafish. These include transgenic strains that use fluorescent protein expression to label cardiomyocytes, for example using the *myl7* promoter (Huang et al., 2003), to visualize asymmetric heart morphogenesis. Similarly, promoters, including sequences from *sox17*, are used to label endoderm to observe gut asymmetries (Chung and Stainier 2008). A searchable catalog of transgenic strains is available at http://zfin.org/action/fish/search. In addition, some asymmetric organ morphologies can be visualized in unlabeled transparent living embryos under a dissecting microscope. These include 1) the direction of heart jogging and looping, which is aided by observing heart contractility and blood flow (Moreno-Ayala et al., 2021) (Supplementary Movie S1), and 2) position of the gall bladder and pancreas that become auto-fluorescent by 7 days post fertilization (Albertson and Yelick 2005; Ji, Buel, and Amack 2016). As a complementary approach to live imaging, several RNA *in situ* hybridization probes are widely available to visualize LR asymmetries in lateral plate mesoderm, heart, brain, gut tube, pancreas, and liver in the zebrafish embryo (Supplementary Table S1). This set of tools provides multiple approaches to visualize and quantify morphometrics of cells and organs, and dissect mechanisms that underlie organ laterality.

### Kupffer’s vesicle is the zebrafish left-right organizer

**FIGURE 4:**
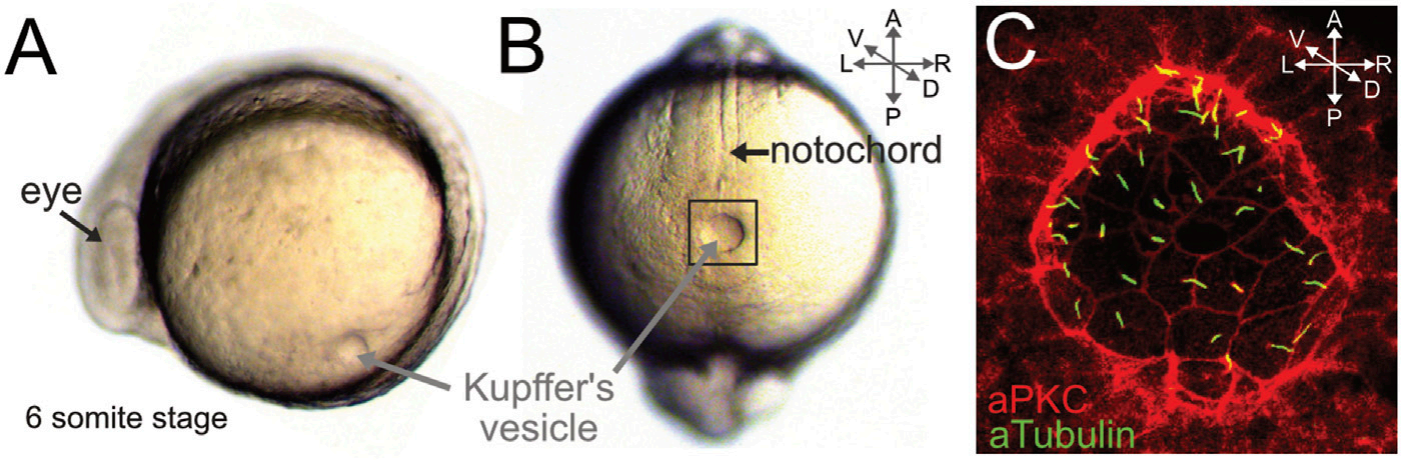
Kupffer’s vesicle is the zebrafish left-right organizer. **(A–B)** Live images of zebrafish embryos at the 6-somite stage (∼12 hpf). Side view **(A)** and dorsal view **(B)** of the fluid-filled Kupffer’s vesicle (KV) lumen in the tailbud. KV is adjacent to the posterior end of the midline notochord. **(C)** Higher magnification of fluorescent immunostaining of KV (boxed region in B) using aPKC antibodies to outline epithelial KV cells (red) and acetylated Tubulin antibodies to label cilia (green). Cilia project b from each KV cell into the lumen. A = anterior, P = posterior, L = left, R = right, D = dorsal, V = ventral.

Work in the mid 2000s identified a transient fluid-filled structure called Kupffer’s vesicle (KV) as the zebrafish LRO. KV forms in the tailbud of the zebrafish embryo during early somitiogenesis and appears as a sphere-like cavity (Figures 4A, B). First described in the 19th century (Kupffer 1868), KV is a conserved transient structure in the teleost embryo. Electron microscopy of killifish (*Fundulus heteroclitus*) embryos revealed a single cilium projecting from each KV cell into the lumen of the cavity (Brummett and Dumont 1978). However, the function of KV remained unknown for over 100 years and it was referred to as an “organ of ambiguity” (Warga and Stainier 2002). Subsequently, the discovery that KV cilia are motile and generate fluid flow inside the KV lumen, along with functional evidence that KV is required for LR patterning (Essner et al., 2002; Amack and Yost 2004; Essner et al., 2005; Kramer-Zucker et al., 2005), revealed KV to be an “organ of asymmetry” that is now referred to as the zebrafish LRO. The zebrafish KV has a simple structure: a single layer of ∼50 ciliated epithelial cells line the fluid-filled lumen (Figure 4C). High-speed videomicroscopy indicates most motile KV cilia beat in a vortical pattern at a frequency of ∼30 rotations per second (∼30 Hz) (Kramer-Zucker et al., 2005; Okabe et al., 2008; Sampaio et al., 2014) (Supplementary Movie S2). These cilia create a directional counterclockwise fluid flow when viewed dorsally (Supplementary Movie S3). Perturbations that disrupt motile KV cilia indicate the directional fluid flow is essential for directing Spaw/Nodal signaling on the left side of the embryo (Essner et al., 2005; Kramer-Zucker et al., 2005; Lopes et al., 2010). Furthermore, mutations that disrupt KV form or function result in laterality defects in zebrafish (reviewed in (Matsui and Bessho 2012; Amack 2014)). Importantly, mechanical disruption or laser ablation of KV cells disrupts LR patterning in the zebrafish embryo without altering dorsal-ventral or anterior-posterior patterning (Essner et al., 2005). This feature, in combination with rapid development and accessibility to high-resolution imaging, make the zebrafish KV a useful model system to investigate LRO biology.

**FIGURE 5:**
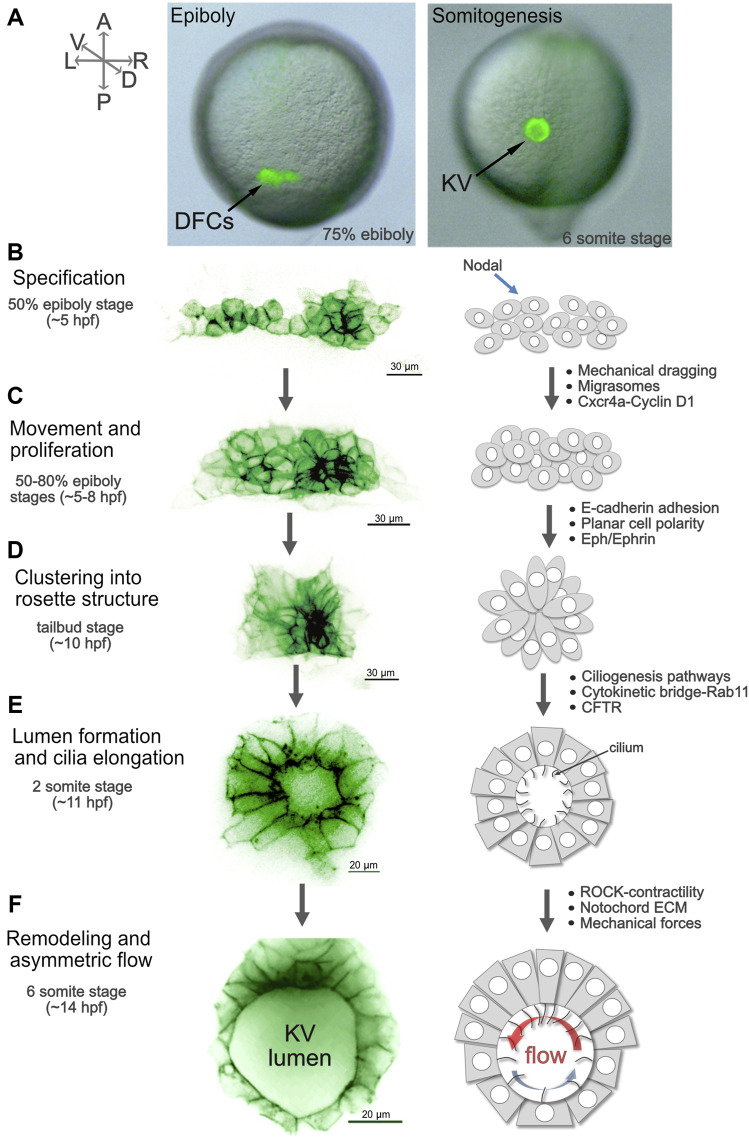
Steps of Kupffer’s vesicle morphogenesis. **(A)** Live images of *Tg* (*sox17:GFP*) (Chung and Stainier 2008) embryos that express GFP (green) in dorsal forerunner cells (DFCs) and Kupffer’s vesicle (KV) show the location of these cells in the zebrafish embryo during epiboly and early somitogenesis. The GFP signal is superimposed on a brightfield image of the embryo. A = anterior, P = posterior, L = left, R = right, D = dorsal, V = ventral. The orientation of the axes shown in **(A)** applies to all images **(A–F)**. **(B–F)** High-resolution snapshots of DFCs and KV cells in live *Tg(sox17:EGFP-CAAX)* (Dasgupta et al., 2018) embryos expressing membrane-localized GFP (shown using an inverted thalium lookup table) alongside simplified diagrams of cell morphologies that represent specific steps of DFC/KV development. Examples of mechanisms that regulate each step are listed (see main text for details). **(B)** Approximately 20–30 mesenchymal DFCs are specified at the 50% epiboly stage. **(C)** DFCs move towards the posterior of the embryo and proliferate between the 50–80% epiboly stages (Supplementary Movie S5). **(D)** At the end of epiboly, DFCs undergo a mesenchymal-to-epithelial transition, and cluster to form a rosette-like structure (Supplementary Movie S6). **(E)** During early somite stages, a fluid-filled lumen expands (Supplementary Movie S6) and cilia elongate into the lumen. At the 2-somite stage anterior and posterior KV cells have similar morphologies. **(F)** Between the 4 and 6 somite stages KV cells undergo asymmetric cell shape changes along the anterior-posterior axis, termed KV remodeling (Supplementary Movie S7). This allows tight packing of cilia in the anterior region of KV to drive strong right-to-left fluid flow (red arow). More widely spaced cilia in the posterior region mediate a slower left-to-right flow (blue arrow).

Several developmental steps that build a functional KV have been identified. The precursor cells that give rise to KV—called dorsal forerunner cells (DFCs)—can be readily tracked and manipulated during development. Transgenic strains have been developed that label the DFC/KV cell lineage, which has facilitated live imaging studies to visualize KV development with high temporal and spatial resolution (Chung and Stainier 2008; Wang et al., 2011; Woo et al., 2012; Dasgupta et al., 2018). DFCs appear at mid-epiboly stages (∼5 hpf), migrate, proliferate, and then undergo a mesenchymal-to-epithelial transition to form KV during early somite stages (Supplementary Movie S4; Figure 5). KV develops directional fluid flow and establishes LR signaling, and then breaks down ∼18 hpf when KV cells undergo a poorly understood epithelial to mesenchymal transition (Amack 2021) and migrate away to incorporate into muscle and notochord (Cooper and D’Amico 1996; Melby, Warga, and Kimmel 1996; Ikeda et al., 2022). While there are certainly species-specific differences in LRO architectures—including overall size, shape, and number of cells (Lee and Anderson 2008; Blum et al., 2009; Amack 2014)—there is a common overarching strategy in several vertebrates to form an LRO that places a mono-ciliated epithelium in a specific geometry that facilitates the creation and detection of directional cilia-driven fluid flow. It will be key to identify mechanisms that control LRO development in several vertebrates in order to construct an evolutionary history of the LRO and determine the underpinnings of LRO form and function.

## Insights from zebrafish into mechanisms that control left-right organizer development

Work in zebrafish has made important contributions to our understanding of the vertebrate LRO and underlying causes of human laterality disorders. Specifically, the zebrafish embryo has provided insights into LRO cell biology, cilia function, and fluid flow dynamics. Here, we provide an overview of specific developmental steps of the zebrafish LRO (KV), and highlight recent findings that shed light on mechanisms that regulate LRO form and function.

### Specification and movement of KV precursor cells

Fate-mapping studies show the KV precursor cells (DFCs) appear on the dorsal side of the embryo as a distinct population of ∼25 cells by the 50% epiboly stage (∼5 hpf) (Cooper and D'Amico 1996; Melby, Warga, and Kimmel 1996) (Figures 5A, B). High-resolution live imaging experiments revealed that DFCs are derived from a set of dorsal surface epithelial cells that ingress beneath the enveloping layer (EVL) of epithelial cells covering the embryo between 4 and 5 hpf (Oteiza et al., 2008). DFCs separate away from deep cells at the blastoderm margin, but some DFCs remain connected to overlying EVL cells *via* contact points that are proposed to seed polarization during later rosette formation. Specification of DFCs is mediated by Nodal signaling (Figure 5B), and gain of function experiments indicate Nodal regulates the ingression of dorsal surface cells to become DFCs (Oteiza et al., 2008). Intriguingly, the number of DFCs during epiboly stages can be quite variable from embryo to embryo, ranging from 10 to 50 cells (Moreno-Ayala et al., 2021). The underlying cause(s) of this variability is not completely understood, but inter-strain crosses and transcriptomics indicate DFC/KV cell number variability correlates with maternal genetic background (Moreno-Ayala et al., 2021). Between 50% and 90% epiboly stages (∼5–9 hpf), when other mesendoderm cells internalize during gastrulation, DFCs move along the surface of the yolk cell ahead of the dorsal margin of the blastoderm towards the vegetal pole of the embryo (Supplementary Movie S5). Movement of DFCs mirrors similar epiboly movements of the overlying EVL cell layer, and by the end of epiboly DFCs form a tight cluster that will give rise to KV.

#### Recent insights

Recent lineage tracing experiments by Warga and Kane have extended our understanding of the origins of DFCs (Warga and Kane 2018). This work indicates DFCs are descendants of specific EVL cells termed “Wilson cells” that are the last cells to share cytoplasmic connections with the yolk cell (up to 512-cell stage) at the blastoderm margin. Wilson cells were found to undergo asymmetric division, with one daughter cell contributing to the yolk syncytial layer, and the other contributing to surface epithelial (EVL) cells. On the dorsal side of the embryo, surface epithelial cells derived from Wilson cells ingress to become DFCs. Consistent with previous reports, DFC specification from Wilson cells depends on Nodal signaling. The Wilson cell origin of DFCs advances our understanding of how LRO cells arise in vertebrate embryos. In addition, this study provides an explanation for previous observations that DFCs remain connected to the yolk cell longer than other cells (Cooper and D'Amico 1996), which has been exploited to deliver synthetic antisense oligonucleotides or mRNAs to modulate gene expression specifically in DFCs (Amack and Yost 2004; Wang, Yost, and Amack 2013; Matsui, Ishikawa, and Bessho 2015).

How DFCs move in a developing embryo to get to the right place at the right time to form KV is not fully understood. A recent report by Pulgar, et al. described a mechanical dragging mechanism that guides DFC movement during epiboly stages (Pulgar et al., 2021). Live imaging of transgenic embryos revealed that DFCs delaminate from dorsal EVL cells using an apical constriction mechanism. In some cells this delamination process is incomplete, which results in physical connections that are maintained between DFCs and overlying EVL *via* apical attachments enriched with junctional proteins. Results from genetic and laser ablation experiments indicated these apical attachments between DFCs and EVL support a mechanism in which spreading of EVL during epiboly can physically drag the attached DFCs towards the posterior pole of the embryo. Strikingly, live imaging of individual DFCs unattached to EVL revealed these cells could migrate away from the DFC group. To follow up on this finding, the authors also identified cell-cell interactions between DFCs—mediated by E-cadherin—that are critical for linking EVL-attached DFCs with unattached DFCs to ensure DFCs move collectively. These results provide insights into mechanisms that connect, organize, and move DFCs during development.

In addition to biophysical dragging, an independent study by Jiang, et al. identified biochemical signaling involved in directing DFC movement during epiboly stages (Jiang et al., 2019). Migrasomes are a recently defined class of extracellular vesicles left at the trailing end of migrating cells that can burst to release signaling molecules (da Rocha-Azevedo and Schmid 2015). Here, the authors identify and characterize migrasomes in the zebrafish embryo. Migrasomes were found to accumulate in a previously unrecognized ‘embryonic shield cavity’ between DFCs and the yolk cell. Mutations that affect the transmembrane proteins Tspan4a or Tspan7 reduced migrasome formation and disrupted movement of the DFC cluster. This resulted in small KVs with a reduced number of ciliated cells. Remarkably, when migrasomes purified from zebrafish embryos were injected into the ventral side of an embryo, DFCs migrated towards the injection site. Mass spectrometry of purified migrasomes identified over 2,000 proteins enriched in migrasomes, including the chemokine Cxcl12a. Genetic experiments indicated the chemokines Cxcl12a/b and the receptor Cxcr4b are required for proper DFC movement and KV development. These results suggest Cxcl12-enriched migrasomes function as chemoattractants that guide DFC movement. Thus, recent work implicates an interplay between biophysical and biochemical mechanisms that direct DFC movement.

### KV precursor cell proliferation, clustering, and lumenogenesis

While moving during epiboly stages, DFCs proliferate at a rate that is higher than non-DFC cells in the embryo (Rathbun et al., 2020). The percentage of DFCs undergoing cell division peaks between 60 and 70% epiboly with a mitotic index between 5 and 10%, and then becomes reduced as mesenchymal DFCs differentiate into ciliated epithelial KV cells (Gokey, Dasgupta, and Amack 2015; Liu et al., 2019; Rathbun et al., 2020). Mechanisms that regulate cell division during KV development—including which cells divide and when—remain poorly understood, but some factors and pathways that impact DFC/KV proliferation have been identified. First, Wnt/β-catenin signaling has been implicated in KV cell division (Caron et al., 2012; Zhang et al., 2012). DFC-specific antisense depletion of Wnt-responsive transcription factors β-catenin-1 or β-catenin-2 reduced the number of KV cells and the size of the KV lumen. Loss of Wnt/β-catenin reduced proliferation of KV cells during lumen formation stages, which led to a smaller lumen size. Second, depletion of the cell surface heparan sulfate proteoglycan Syndecan 2 reduced the number of mitotic DFCs, resulting in fewer KV cells (Arrington, Peterson, and Yost 2013). Rescue experiments indicated that Syndecan 2 cooperates with Fgf2 to regulate DFC proliferation and KV morphogenesis. Third, antisense depletion or pharmacological inhibition of the vacuolar-ATPase (V-ATPase) proton pump reduced the mitotic index of DFCs during the 60–90% epiboly stages (Gokey, Dasgupta, and Amack 2015). This reduced the number of KV cells, decreased KV size and altered embryo laterality. V-ATPase has been implicated in laterality for many years (Adams et al., 2006), and may impact DFC proliferation by regulating signaling pathway(s) that include mTOR, Notch, and Wnt (Sun-Wada and Wada 2015). It is interesting to note that in each of these studies defects in proliferation were associated with short KV cilia, suggesting co-regulation of cell cycle and cilia length during KV development. Interestingly, depletion of the centrosomal protein Nde1, which reduced DFC proliferation, increased KV cilia length (Kim et al., 2011). Taken together, these findings suggest a complex coordination between DFC/KV cell proliferation and ciliogenesis that remains poorly understood.

The initially loosely associated group of DFCs forms a tight cluster of cells by the 80% epiboly stage (∼8 hpf) (Figure 5C; Supplementary Movie S5). Defects in this clustering process disrupt KV formation, and several genes and pathways have been implicated in this process. These include E-cadherin (Cdh1) based cell-cell adhesion (Shibasaki et al., 1996), the transcription factor Tbx16 (Amack, Wang, and Yost 2007; Matsui et al., 2011), Ca^2+^ signaling (Schneider et al., 2008; Lai et al., 2012), integrin-extracellular matrix interactions (Ablooglu et al., 2010), planar cell polarity signaling (Oteiza et al., 2010), the actin capping protein Arp2/3 myosin-I linker CARMIL3 (Stark et al., 2022), and FGF signaling (Matsui et al., 2011). In addition, Eph/Ephrin signaling was found to function as a boundary that keeps DFCs from clustering with other cell types (Zhang et al., 2016). Details of mechanisms that regulate DFC clustering have been previously reviewed (Matsui, Ishikawa, and Bessho 2015). At the end of epiboly (∼9 hpf), the advancing blastoderm overtakes the DFCs, which now detach from the EVL and move deep into the embryo still adjacent to the yolk cell. At the tailbud stage (∼10 hpf) DFCs rearrange to form a rosette-like structure around foci of apical membrane proteins (e.g., aPKC, ZO1) as the mesenchymal DFCs polarize and transition into epithelial KV cells (Amack, Wang, and Yost 2007; Oteiza et al., 2008; Navis, Marjoram, and Bagnat 2013) (Figure 5D). A nascent fluid-filled lumen begins to form at the center of the rosette structure, which then expands rapidly (Oteiza et al., 2008; Navis, Marjoram, and Bagnat 2013) (Figure 5E; Supplementary Movie S6). Expansion of the lumen is thought to proceed using a combination of vacuole-like structures that fuse with the apical membrane of KV cells (Oteiza et al., 2008; Saydmohammed et al., 2018), and ion transporters that drive transepithelial water flow (Navis, Marjoram, and Bagnat 2013; Dasgupta et al., 2018). One of these ion channels, the cystic fibrosis transmembrane conductance regulator (Cftr), is expressed in KV during early embryogenesis, and mutation of *cftr* blocks lumen expansion (Navis, Marjoram, and Bagnat 2013). Finally, maintaining the integrity of junctions between KV cells is also critical for lumen expansion (Kim et al., 2017; Dasgupta et al., 2018). Consistent with variable numbers of DFCs discussed above, the number of KV cells and KV lumen size can be quite variable in a population of wild-type embryos. There are typically ∼50 ciliated cells in KV at the 8 somite stage, but this number ranges significantly between 20 and 90 cells (Gokey et al., 2016; Moreno-Ayala et al., 2021). Mechanisms underlying this variability are not understood, but analyses of single embryos suggest exceeding a threshold of KV size is needed for reliable LR patterning (Gokey et al., 2016).

#### Recent insights

A recent study by Liu, et al. provided new molecular insights that connect cell proliferation and ciliogenesis during KV development (Liu et al., 2019). This work identified new functions for chemokine signaling—in addition to the roles in migrasome-mediated DFC movement described above—in regulating DFC proliferation and KV cilia length. Loss-of-function mutations in the chemokine receptor *cxcr4a* resulted in fewer KV cells, shorter KV cilia, and laterality defects. Genetic and biochemical studies indicated Cxcr4a signaling, mediated by an ERK1/2 pathway, activates Cyclin D1 expression in DFCs to stimulate G1/S progression during the cell cycle. In addition, Cyclin D1 was found to stabilize the transcription factor Foxj1a, a known regulator of genes that build motile cilia. The Cyclin D1-associated Cyclin-dependent kinase 4 (Cdk4) was then shown to interact with and phosphorylate Foxj1a. This phosphorylation event prevented Foxj1a degradation to promote ciliogenesis. Thus, Cxcr4a signaling *via* Cyclin D1 regulates both cell cycle progression and ciliogenesis during KV development.

Using high-resolution live imaging and novel optical approaches, Rathbun, et al. identified a mechanistic link between cell division and KV lumenogenesis (Rathbun et al., 2020). Live imaging revealed that after DFC mitotic division, the two daughter cells remain connected *via* a cytokinetic bridge that becomes positioned at the center of rosette structures where KV lumens form. These bridges are then cleaved in the last stage of mitosis called abscission. Premature severing of DFC cytokinetic bridges using laser ablation was found to greatly reduce KV lumen size, suggesting an essential role for cytokinetic bridges in lumen formation. To block cytokintetic bridge severing, the authors developed an optogenetic approach targeting the GTPase Rab11, which is known to be present on endosomes necessary for the initiation of abscission, and has previously been implicated in KV lumen formation (Westlake et al., 2011; Tay et al., 2013). Light-mediated clustering of Rab11 + endosomes prevented abscission and blocked KV lumen formation. Furthermore, Cftr protein, which is needed for lumen expansion (Navis, Marjoram, and Bagnat 2013), was found to co-localize with clustered Rab11+ vesicles and failed to localize to apical membranes of KV cells. These results suggest a mechanism in which Rab11+ vesicles traffic Cftr, and potentially other proteins, to cytokintetic bridges that give rise to apical membranes of KV cells to mediate lumen formation and expansion.

### KV cilia and flow dynamics

During lumen formation, each KV cell projects a cilium from its apical surface into the expanding lumen. Cilia genes are expressed early in DFCs, and make good markers for the DFC/KV cell lineage since KV is the first ciliated organ to form in the zebrafish embryo. KV cilia elongate between the tailbud stage and 8 somite stage to reach a final length of ∼5 microns, which is similar to other vertebrate LRO cilia (Amack, Wang, and Yost 2007; Oteiza et al., 2008; Gokey et al., 2016). Proper KV cilia formation and length, which is critical for generating effective fluid flow and LR patterning (Pintado et al., 2017), is influenced by several signaling pathways. These include FGF (Hong and Dawid 2009; Neugebauer et al., 2009; Qian et al., 2013), Wnt (Oishi et al., 2006; Schneider et al., 2010; Caron et al., 2012), TOR (Yuan et al., 2012; Burkhalter et al., 2013), Laminin/extracellular matrix (Hochgreb-Hagele et al., 2013), prostaglandin (Jin et al., 2014; Chambers et al., 2020), and Notch (Lopes et al., 2010) signaling. In addition, molecules that are known regulators of vesicle trafficking—the BBSome (Yen et al., 2006; May-Simera et al., 2010; Veleri et al., 2012), V-ATPase (Chen et al., 2012; Gokey, Dasgupta, and Amack 2015), and Rab GTPases (Kuhns et al., 2019)–-have been implicated in KV ciliogenesis. Finally, several proteins have been identified as essential for KV cilia motility and zebrafish LR asymmetry, and in some cases KV has been used effectively as a model system to study human mutations in cilia genes identified in patients with laterality defects (Becker-Heck et al., 2011; Jerber et al., 2014; Narasimhan et al., 2015; Li et al., 2016; Noel et al., 2016; Yamaguchi et al., 2018; Burkhalter et al., 2019; Sasaki et al., 2019; Xie et al., 2019).

Similar to mouse and frog LRO cilia, AP polarization of KV cilia is regulated by planar cell polarity (Borovina et al., 2010). In addition, cilia were found to have different orientations on the left and right sides of KV, which is mediated by planar cell polarity and cilia motility (Ferreira et al., 2018). Many KV cilia are immotile during early somites stages (∼30–40% are immotile between 1 and 4 somite stages), whereas nearly all cilia (∼98%) are motile by the 8-somite stage (Yuan et al., 2015; Ferreira et al., 2017). Several methods have been developed to quantify the dynamics of fluid flows generated by motile cilia in the KV lumen. These include tracking injected fluorescent microspheres (Essner et al., 2005; Wang, Yost, and Amack 2013; Fox, Manning, and Amack 2015), laser-generated cellular debris (Supatto, Fraser, and Vermot 2008), and naturally occurring particles (Sampaio et al., 2014). Quantification of flow velocities in different regions of KV revealed higher velocities in the anterior region as compared to the posterior region (Wang et al., 2011; Sampaio et al., 2014). This results in a strong leftward flow across the anterior pole of KV, and a slower rightward return flow across the posterior end (Figure 5F; Supplementary Movie S3). The left-anterior KV, which experiences the strongest flow, is the region where the first molecular asymmetries (Ca^2+^ signals and activation of CaMKII discussed below) are detected (Francescatto et al., 2010; Yuan et al., 2015).

#### Recent insights

Two studies have uncovered roles for the actin-dependent myosin motor protein Myo1d in KV development and LR patterning in the zebrafish embryo (Juan et al., 2018; Saydmohammed et al., 2018). Myo1d was first identified in genetic screens as a regulator of LR asymmetry in the invertebrate *Drosophila melanogaster* (Hozumi et al., 2006; Speder, Adam, and Noselli 2006), which does not use cilia to break bilateral symmetry. Myo1d interacts with the planar cell polarity (PCP) pathway to mediate asymmetric looping of the hindgut in *Drosophila* (Gonzalez-Morales et al., 2015). In zebrafish *myo1d* mutants generated by Juan, et al., KV lumen was often reduced in size and contained fewer and shorter cilia (Juan et al., 2018). Quantification of fluid flow dynamics inside KV revealed reduced angular velocity in mutants, which correlated with LR patterning defects. Myo1d was found to genetically interact with the PCP protein Vangl2 to control the polarization of cilia in KV that is necessary for robust directional flow. Separate work in *Xenopus* indicates Myo1d interacts with Vangl2 to regulate PCP signaling, LRO cilia orientation, asymmetric fluid flow, and LR patterning in frog embryos (Tingler et al., 2018). A second study in zebrafish by Saydmohammed, et al. using independently generated mutations in *myod1* reported similar phenotypes, including reduced KV lumen size, fewer KV cilia, disrupted KV flow dynamics, and laterality defects (Saydmohammed et al., 2018). Here, the authors identified a role for Myo1d in mediating the transport of fluid-filled vesicles (or vacuoles) that fuse with the apical plasma membrane to contribute to lumen expansion. Together, these results indicate Myo1d is a key player in establishing fruit fly, zebrafish, and frog LR asymmetry, which provides an evolutionarily conserved link between invertebrate and vertebrate laterality.

As a complementary approach to biological experiments, mathematical models have provided important insights into fluid dynamics inside KV. Computational modeling of KV flow presented by Ferreira, et al. (Ferreira et al., 2017) indicated the flow dynamics observed *in vivo* could be generated by cilia tilted towards the meridian of the spherical KV. To test this prediction, live imaging of KV in wild-type embryos was used to create a 3-dimensional map of average spatial distribution, motility, and orientation of KV cilia. Measurements of cilia orientation revealed the majority of motile cilia (∼65%) indeed have a meridional tilt, suggesting this orientation is a key mechanism that drives unidirectional flow. Simulations of flow dynamics based on *in vivo* cilia properties predicted that the number of immotile cilia is too small to reliably detect flows inside KV *via* mechanosensation. On the other hand, modeling suggested flow could asymmetrically transport secreted particles larger than 2 nm, which is in the size range of extracellular vesicles. Mathematical models of KV flow dynamics from an independent group (Solowiej-Wedderburn et al., 2019) suggested that mechanical stress on the plasma membrane generated by motile KV cilia may promote the release of extracellular vesicles. Release of extracellular vesicles in the anterior pole of KV would be transported to the left side to potentially mediate chemosensing of flow. An alternative possibility supported by simulations is that cells sense movement of their own cilia (Ferreira et al., 2017), which could be mediated by mechano- or chemo- sensing mechanisms (Cartwright, Piro, and Tuval 2020).

### KV remodeling, KV cellular architecture, and LR signaling

LROs in vertebrate embryos develop a specific cellular architecture that is critical for generating fluid flow and LR asymmetry. The zebrafish KV at the 8 somite stage has an asymmetric architecture along the AP axis, such that more ciliated cells are placed in the anterior region, and fewer are positioned in the posterior pole (Kreiling et al., 2007; Okabe et al., 2008; Ferreira et al., 2017). This AP gradient of cilia is due to regional differences in cell shape: cells in the anterior region are elongated and tightly packed together, and cells in the posterior region are cuboidal and spread further apart (Wang et al., 2011). Inhibiting these cell shape changes disrupts the asymmetric cilia distribution, eliminates directional flow in KV, and causes laterality defects (Wang, Manning, and Amack 2012). These results, along with computational modeling results (Montenegro-Johnson et al., 2016), indicate anterior clustering of cilia is critical for generating the observed asymmetric flow in KV: stronger right-to-left flow in the anterior region, and a weaker flow from left-to-right in the posterior region. This strong leftward flow in the anterior KV is proposed to be analogous to leftward flow in the mouse LRO.

Live imaging determined that changes in KV cellular architecture occur with very precise developmental timing. At the 2-4 somite stages (12–13 hpf), all cells at the middle plane of the KV have similar cell shapes (Figure 5E). However, by the 6-somite stage (14 hpf), anterior KV cell shapes are morphologically different from posterior cells, and this difference persists until at least the 10-somite stage (Wang, Manning, and Amack 2012) (Figure 5F). We refer to this process of differential cell shape changes along the AP aixs as KV remodeling (Supplementary Movie S7). Several mechanisms have been identified that contribute to KV remodeling. First, treatments that alter contractility of the actomyosin cytoskeleton blocked KV remodeling and LR patterning without affecting lumen expansion or cilia motility (Wang et al., 2011; Wang, Manning, and Amack 2012). Results from antisense depletion of the Rho kinase *rock2b* specifically in KV cells suggests a cell autonomous function for contractility during KV remodeling. Genome-wide analyses in patients with laterality defects identified variants in *ROCK2* (Fakhro et al., 2011; Li et al., 2019), implicating Rho kinase activity in human laterality. Second, the midline notochord structure, which is positioned adjacent to the anterior and dorsal regions of the KV, has a key role in KV remodeling (Compagnon et al., 2014). In innovative experiments, activation of Nodal signaling was used to create ectopic clusters of DFCs that formed KVs in random regions of the embryo. Strikingly, these ectopic KVs could undergo remodeling, but only if ectopic notochord tissue was co-induced with the KV. Additional experiments revealed that the notochord deposits extracellular matrix (ECM) components, including Laminin and Fibronectin, that accumulate at the anterior region of KV and are necessary for KV cell shape changes to occur. Third, analyses of 3D renderings of single KV cells revealed cell shape changes similar to those identified in 2D studies, and also uncovered asymmetric cell volume changes: anterior cells increase in size, whereas posterior cells decrease in size (Dasgupta et al., 2018). Cell volume changes were found to be regulated by ion flux mediated by sodium-potassium pump (Na+/K+-ATPase) activity and Cftr. Another likely mechanism regulating cell volume is Myo1d, which traffics vacuoles—particularly in posterior KV cells—to the apical membrane during lumen expansion (Saydmohammed et al., 2018). Possible interactions between actyomyosin contractility, ECM, and/or cell volume changes that may converge to regulate KV cell shapes await further investigation.

LR signaling at KV is not fully understood. As in other vertebrate embryos, there is asymmetric Ca^2+^ flux on the left side of KV during early somite stages (Sarmah et al., 2005; Jurynec et al., 2008; Yuan et al., 2015). The presence of immotile cilia in KV—albeit randomly distributed (Sampaio et al., 2014; Yuan et al., 2015)—supports a model for mechanosensory cilia detecting directional flow and triggering LR asymmetric signaling cascades. Recent work indicates the number of immotile cilia is controlled by Notch signaling (Tavares et al., 2017). In addition, stretch-activated PKD cation channels, composed of Pkd2 and Pkd1l1 subunits, localize to KV cilia and are necessary for LR pattering (Kamura et al., 2011; England et al., 2017). High-resolution imaging of genetically encoded Ca^2+^ indicators targeted to KV cilia detected Ca^2+^ fluxes called intraciliary calcium oscillations (ICOs) that initiate in KV immotile cilia and then propagate as cytoplasmic Ca^2+^ fluxes in surrounding cells (Yuan et al., 2015). These ICOs depend on motile cilia-generated fluid flow in KV and the cation channel Pkd2. Interestingly, ICOs peak between 1 and 4 somite stages when most cilia are immotile and robust directional flow has not yet been established. An asymmetric bias of ICOs on the left side of KV is proposed to impact downstream *dand5* and *spaw* expression. Beginning at the 3-somite stage, the Ca^2+^-dependent protein kinase CaMKII was found be transiently activated in KV cells (Francescatto et al., 2010). Asymmetric activation (phosphorylation) of CaMKII in left-anterior KV cells peaked between 10 and 12 somite stages, suggesting CaMKII may be a target of Ca^2+^ fluxes initiated by ICOs. However, a connection between these events has not been established.

#### Recent findings

Regulation of KV architecture has recently by linked to molecules involved in other steps of LR patterning. First, Jacinto, et al. (Jacinto et al., 2021) analyzed KV architecture in *pkd2* mutants, called *curly up* (*cup*) (Schottenfeld, Sullivan-Brown, and Burdine 2007), and found defects in posterior KV cell shape changes that correlated with faster KV flow dynamics. The authors suggest that KV architecture defects in *cup* mutants could impact flow by altering the spacing between posterior KV cilia. Second, a recent study by Pelliccia, et al. (Pelliccia, Jindal, and Burdine 2017) implicates TGF-β/Nodal signaling, which is required for DFC specification and left-sided signaling in lateral plate mesoderm, in regulating KV cell shapes by studying the Nodal co-factor Gdf3. Maternal and zygotic *gdf3* (*mzgdf3*) mutants develop pleiotropic developmental defects caused by reduced nodal signaling that are too severe to score LR asymmetry (Bisgrove et al., 2017; Pelliccia, Jindal, and Burdine 2017). However, partial rescue of *mzgdf3* embryos with *gdf3* mRNA injections uncovered LR patterning defects (Pelliccia, Jindal, and Burdine 2017). Antisense depletion of maternal and zygotic Gdf3 altered KV cell shapes, disrupted *dand5* asymmetry, and abolished *spaw* expression in lateral plate mesoderm. This suggests Gdf3 mediates KV remodeling that is necessary for generating fluid flow and downstream LR asymmetry. However, in a separate study by Peterson, et al., antisense depletion of Gdf3 (also called Dvr1) was found to attenuate *spaw* in lateral plate mesoderm, but had no measurable effect on fluid flow in KV (Peterson, Wang, and Yost 2013). These phenotypic differences in KV, which may be due to different Gdf3 knockdown levels, potentially point to the sensitivity of TGF-β/Nodal signaling to co-factor availability.

In addition to signaling pathways, biophysical forces have been recently implicated in mediating cell shape changes during KV remodeling. During KV remodeling stages KV is not static, but rather moves posteriorly in the tailbud of the elongating embryo. Erdemci-Tandogan, et al. (Erdemci-Tandogan et al., 2018) determined that KV moves faster than the surrounding tailbud cells during these stages. Mathematical modeling in this study predicts that KV moving through the tailbud tissue creates drag forces on KV that could generate the cell shape changes observed *in vivo*. Specifically, the model predicts these drag forces depend on the rate of movement of KV and fluid-like properties of the tailbud tissue. In follow up work, Sanematsu, et al. (Sanematsu et al., 2021) developed a 3D model of KV moving through tailbud tissue to further characterize drag forces on KV. 3D simulations and quantitative analyses of KV movement relative to tailbud cells in live embryos indicated KV experiences drag forces that contribute to KV cell shape changes. Additional work is needed to experimentally test the predictions of these models and determine the impact of drag forces on KV morphogenesis.

## Conclusion and future directions

Errors during early embryogenesis that disrupt the development and arrangement of internal organs along the LR body axis can result in a wide spectrum of birth defects and associated health problems. Research during the last several decades has made tremendous progress towards understanding the underlying biology of laterality defects. It is now clear that an evolutionarily conserved Nodal signaling cascade is asymmetrically activated at a transient left-right organizer to establish distinct left and right sides in developing vertebrate embryos. However, a number of questions remain about the mechanisms that generate appropriate asymmetric Nodal signals in vertebrate embryos, and how molecular asymmetries are translated in morphological asymmetries. Several animal models have played key roles in elucidating *in vivo* features and regulators of the LR patterning process. Among these, zebrafish has provided important insights into mechanisms that control the construction of a functional LRO.

The rapid advancement of high-resolution *in vivo* imaging approaches to visualize and quantify cell behaviors, fluid flows, and Ca^2+^ dynamics puts zebrafish in position to address open questions about the earliest steps of LR development. These include when, what, and where is the first break in bilateral symmetry? Previous work in the frog *Xenopus leavis* suggested the first molecular LR asymmetries appear at cleavages stages during very early development (Levin et al., 2002; Fukumoto, Kema, and Levin 2005; Adams et al., 2006), and more recent single cell mass spectrometry studies uncovered metabolic differences between left and right blastomeres in the 8-cell *Xenopus* embryo that may influence LR patterning (Onjiko et al., 2016; Onjiko, Nemes, and Moody 2021). However, the role(s) for cleavage-stage asymmetries versus the ciliated LRO during symmetry breaking has been controversial (Levin and Palmer 2007; Schweickert et al., 2012; Vandenberg, Lemire, and Levin 2013; Blum et al., 2014). Whether molecular and/or cellular asymmetries develop prior to LRO function in zebrafish remains unknown. In addition, it will be important to discover new mechanisms that link observed molecular LR asymmetries at the LRO—such as fluid flow, ICOs, activated CaMKII, and *dand5*—to a linear pathway and/or parallel/alternative pathways. Zebrafish embryos are also poised to provide a test ground for investigating how interplay between biochemical signaling and biophysical forces regulate LRO development, and perhaps organ morphogenesis more broadly. Continued development of mathematical models, in parallel with new laser ablation and optogenetic approaches, are predicted to produce more sophisticated studies of physical forces during LRO development. Lastly, advances in gene editing technologies will allow zebrafish to be used rapidly and effectively to test candidate genes produced by large-scale sequencing projects in human patients. Rapid external development, combined with useful transgenic tools already in hand, makes the zebrafish an attractive system to develop high-throughput pipelines for screening gene mutations. Advances along these fronts are expected to uncover new cellular, molecular, and biophysical mechanisms underlying normal and abnormal organ development, which may ultimately improve prediction, diagnosis, and management of human laterality disorders.

## Supplementary material

Supplementary Movie S1 Visualization of a normal rightward looped heart in a wild-type zebrafish embryo at 2 days post-fertilization. V = ventricle; A = atrium. Time scale = minutes:seconds.

Supplementary Movie S2 Motile cilia in KV. Dorsal view of a wild-type embryo showing KV cilia beating in a vortical pattern at the 8-somite stage. The movie was captured at 117 frames per second (fps) and is played back at 10 fps**.** Time scale = hours:minutes:seconds:miliseconds.

Supplementary Movie S3 Fluid flow in KV. Fluorescent microspheres were injected into a live wild-type embryo at the 6-somite stage. The brightfield channel shows KV lumen and the fluorescent channel shows microspheres moving with a directional counterclockwise fluid flow. Note a strong leftward flow in the anterior region of KV, and a slower rightward return flow in the posterior region. Dorsal view. Anterior is at the top.

Supplementary Movie S4 Development of the DFC/KV cell lineage. Live timelapse imaging of a *Tg* (*dusp6:memGFP*) (Wang et al. 2011) embryo expressing membrane-localized GFP in DFC/KV cells from the 90% epiboly stage to the 2 somite stage. The GFP signal is superimposed on a brightfield image of the embryo. During the timelapse, clustered DFCs undergo a mesenchymal-to-epithelial transition to differentiate into epithelial KV cells that form a rosette-like structure. Next, a fluid-filled lumen begins to expand at the center of the forming KV structure. Note that the DFC/KV cells are highly dynamic and continually moving in the embryo. Time scale = hours:minutes:seconds.

Supplementary Movie S5 Cell behaviors of DFCs. Live timelapse imaging of a *Tg* (*sox17:GFP-CAAX*) (Dasgupta et al. 2018) embryo expressing membrane-localized GFP in DFCs (shown using an inverted thalium lookup table) captured DFC movement, proliferation, and clustering from the 60% epiboly stage to the 80% epiboly stage. The embryo was imaged in the chorion. Time scale = hours:minutes.

Supplementary Movie S6 KV rosette-like structure and lumenogenesis. Live timelapse imaging of a *Tg* (*sox17:GFP-CAAX*) (Dasgupta et al. 2018) embryo expressing membrane-localized GFP in KV cells (shown using an inverted thalium lookup table) between tailbud stage and the 4-somite stage. KV lumen expansion initiates at the center of the rosette-like structure. The embryo was imaged in the chorion. Time scale = hours:minutes.

Supplementary Movie S7 KV remodeling. Live timelapse imaging of a double transgenic embryo expressing *Tg* (*sox17:DsRed*) (Chung and Stainier 2008) that labels KV cells (shown in red) and *TgBAC* (*cdh2:cdh2-sfGFP-TagRFP,crybb1:ECFP*) (Revenu et al. 2014) that labels Cadherin2 at cell-cell junctions (shown in green) between the 4 and 6 somite stages. To adjust for KV movement, the movie was edited to place KV in the center for each timepoint. During this 1 h of morphogenesis, anterior and posterior KV cells undergo distinct cell shape changes. Time scale = hours:minutes:seconds.

Supplementary Table S1 Useful molecular markers to visualize LR asymmetries in the zebrafish embryo using RNA *in situ* hybridization.
