# Supplementary material for: Understanding laterality disorders and the left-right organizer: Insights from zebrafish
Source: Front Cell Dev Biol. 2022 Dec 23;10:1035513. doi: 10.3389/fcell.2022.1035513 (PMC9816872; doi:10.3389/fcell.2022.1035513)
Supplement: Supplementary file 1 [file Table1.docx]

**Table S1:** Useful molecular markers to visualize LR asymmetries in the zebrafish embryo using RNA *in situ* hybridization.

| Embryonic  Location | Molecular  Marker | LR  Asymmetry | Developmental  Stage | Reference |
| --- | --- | --- | --- | --- |
| LRO/  Kupffer’s vesicle | *dand5*  *(charon)* | Elevated right-sided expression | 8 SS | (Hashimoto et al. 2004) |
|  |  |  |  |  |
| Lateral plate mesoderm and heart field | *Spaw*  *(nodal-related)* | Left-sided  expression | 10 SS | (Long, Ahmad, and Rebagliati 2003) |
|  | *pitx2* | Left-sided  expression | 22 SS | (Essner et al. 2000)  (Campione et al. 1999) |
|  | *Cyclops*  *(nodal-related)* | Left-sided  expression | 19 SS | (Rebagliati et al. 1998)  (Sampath et al. 1998) |
|  | *lefty1* | Left-sided  expression | 19 SS | (Bisgrove, Essner, and Yost 1999)  (Thisse and Thisse 1999) |
|  | *lefty2* | Left-sided  expression | 19 SS | (Bisgrove, Essner, and Yost 1999)  (Thisse and Thisse 1999) |
|  | *elovl6* | Left-sided  expression | 18 SS | (Ji, Buel, and Amack 2016) |
|  | *prrx1a* | Right-sided expression | 18 SS | (Ocana et al. 2017) |
| Heart field | Bmp4 | Elevated left-sided expression | 22 SS | (Chen et al. 1997) |
|  |  |  |  |  |
| Heart tube | *myl7*  *(cmlc2)* | Leftward jogging | 22-26 hpf | (Yelon, Horne, and Stainier 1999) |
|  |  | Rightward  looping | 28-48 hpf |  |
|  | *nkx 2.5* | Leftward jogging | 22-26 hpf | (Chen and Fishman 1996) |
|  |  | Rightward  looping | 28-48 hpf | (Andre et al. 2000) |
|  |  |  |  |  |
| Gut | *foxa3*  *(fkd2)* | Gut tube asymmetry | 48 hpf | (Odenthal and Nusslein-Volhard 1998) |
|  | *sox17* | Gut tube asymmetry | 48 hpf | (Alexander and Stainier 1999) |
|  | *prox1* | liver | 72 hpf | (Ober, Field, and Stainier 2003) |
|  | *fabp2*  *(ifabp)* | intestine | 72 hpf | (Andre et al. 2000) |
|  | *insulin* | Pancreatic islet | 72 hpf | (Milewski et al. 1998) |
|  | *prss1*  *(trypsin)* | Exocrine pancreas | 72 hpf | (Lin et al. 2004) |
|  |  |  |  |  |
| Brain | cyc | Diencephalon | 19 SS | (Rebagliati et al. 1998)  (Sampath et al. 1998) |
|  | *pitx2* | Diencephalon | 22 SS | (Essner et al. 2000)  (Campione et al. 1999) |
|  | *lefty1* | Habenula | 19 SS | (Bisgrove, Essner, and Yost 1999)  (Thisse and Thisse 1999) |
|  | *lefty2* | Habenula | 19 SS | (Bisgrove, Essner, and Yost 1999)  (Thisse and Thisse 1999) |
|  | *lov* | Habenula | 40 hpf | (Gamse et al. 2003) |
